# Cerebrospinal fluid lipocalin 2 as a novel biomarker for the differential diagnosis of vascular dementia

**DOI:** 10.1038/s41467-020-14373-2

**Published:** 2020-01-30

**Authors:** Franc Llorens, Peter Hermann, Anna Villar-Piqué, Daniela Diaz-Lucena, Katarina Nägga, Oskar Hansson, Isabel Santana, Matthias Schmitz, Christian Schmidt, Daniela Varges, Stefan Goebel, Julien Dumurgier, Henrik Zetterberg, Kaj Blennow, Claire Paquet, Inês Baldeiras, Isidro Ferrer, Inga Zerr

**Affiliations:** 1 Department of Neurology, University Medical Center Göttingen, Göttingen, Germany; 2 Network center for biomedical research of neurodegenerative diseases (CIBERNED), Institute Carlos III, Ministry of Health, Llobregat, Spain; 3 Bellvitge Biomedical Research Institute (IDIBELL), Hospitalet de Llobregat, Llobregat, Spain; 4 Department of Acute Internal Medicine and Geriatrics, Linköping University, Linköping, Sweden; 5 Memory Clinic, Skåne University Hospital, Malmö, Sweden; 6 Clinical Memory Research Unit, Department of Clinical Sciences, Lund University, Malmö, Sweden; 7 Neurology Department, CHUC—Centro Hospitalar e Universitário de Coimbra, CNC- Center for Neuroscience and Cell Biology; Faculty of Medicine, University of Coimbra, Coimbra, Portugal; 8 German Center for Neurodegenerative Diseases (DZNE), Göttingen, Germany; 9 Department of Neurology, Center for Head- and Neuro-Medicine, Klinikum Kassel, Kassel, Germany; 10 Center of Cognitive Neurology and Inserm U942 Lariboisière Hospital AP-HP University Paris Diderot, 75010 Paris, France; 11 Department of Neurodegenerative Disease, Institute of Neurology, University College London, London, UK; 12 UK Dementia Research Institute at UCL, London, UK; 13 Clinical Neurochemistry Laboratory, Sahlgrenska University Hospital, Mölndal, Sweden; 14 Department of Psychiatry and Neurochemistry, Institute of Neuroscience and Physiology, The Sahlgrenska Academy at the University of Gothenburg, Mölndal, Sweden; 15 Department of Pathology and Experimental Therapeutics, University of Barcelona, Hospitalet de Llobregat, Spain; 16 Institute of Neurosciences, University of Barcelona, Barcelona, Spain

**Keywords:** Biomarkers, Neurology, Alzheimer's disease, Neurovascular disorders

## Abstract

The clinical diagnosis of vascular dementia (VaD) is based on imaging criteria, and specific biochemical markers are not available. Here, we investigated the potential of cerebrospinal fluid (CSF) lipocalin 2 (LCN2), a secreted glycoprotein that has been suggested as mediating neuronal damage in vascular brain injuries. The study included four independent cohorts with a total *n* = 472 samples. LCN2 was significantly elevated in VaD compared to controls, Alzheimer’s disease (AD), other neurodegenerative dementias, and cognitively unimpaired patients with cerebrovascular disease. LCN2 discriminated VaD from AD without coexisting VaD with high accuracy. The main findings were consistent over all cohorts. Neuropathology disclosed a high percentage of macrophages linked to subacute infarcts, reactive astrocytes, and damaged blood vessels in multi-infarct dementia when compared to AD. We conclude that CSF LCN2 is a promising candidate biochemical marker in the differential diagnosis of VaD and neurodegenerative dementias.

## Abstract (web summary)

Diagnosis of vascular dementia is hampered by the lack of biochemical markers for this disease. Here, the authors show that vascular dementia is associated with increased lipocalin-2 in cerebrospinal fluid, compared to controls and patients with other forms of dementia.

## Introduction

Vascular dementia (VaD) is considered one of the most common causes of dementia after Alzheimer’s disease (AD)^1^. Identifying VaD patients for epidemiological or clinical research and clinical trials as well as monitoring of therapeutic interventions in VaD trials is challenging. Despite the importance of the disease, diagnosis has been hampered by the lack of well-defined standardized criteria. Commonly used criteria^2^ have suboptimal diagnostic accuracy^3,4^. Unified neuropathological criteria have not been available^5^ in the past and recently published guidelines^6^ have not yet been widely adopted. A research framework for AD proposed in 2018^7^ includes cerebrospinal fluid (CSF) and imaging markers of tau and amyloid-pathology. Recent efforts to standardize and harmonize diagnosis in VaD have led to a consensus study on vascular cognitive impairment (VCI)^8^ and the suggestion of imaging and neuropsychological criteria. However, no specific fluid marker for VaD has been established in the clinical routine to date. Imaging markers related to vascular brain injury (VBI), e.g., white matter hyperintensities (WMH) on magnetic resonance imaging (MRI) are accepted markers for VaD^9,10^ and provide valuable information about cerebrovascular pathology in morphological terms, but they are not pathognomonic^10^. The nature of their association with dementia has not been fully clarified and further research is still needed^11–13^. Mixed forms of dementia (such as VaD plus AD) are frequent and differentiation from pure forms of AD and VaD is not always possible^14^. In addition, WMH are frequent in patients with AD^15^ and might be caused by neurodegeneration^16^. In summary, new biomarkers are needed to improve diagnosis, to uncover the pathophysiology, and to monitor clinical trials^17–20^.

Lipocalin 2 (LCN2) is a secreted glycoprotein involved in innate immunity and highly expressed in the central nervous system in response to injury and inflammatory stimuli^21^. It has been discussed as a potential marker for AD^22^. Studies in humans have shown slightly elevated levels of LCN2 in plasma of patients with mild cognitive impairment (MCI)^23^ and in CSF of patients with multiple sclerosis^24^. LCN2 has also been discussed as an attractive blood-based biomarker of inflammation, ischemia^25^, and, in particular kidney injury^26,27^. Experimental models of VaD have shown that LCN2 mediates hippocampal damage and that LCN2 deficiency is associated with less white matter damage and cognitive decline^28^ but LCN2 has not been clinically evaluated in the context of differential diagnosis of VaD.

The primary aim of the study was to investigate the potential of LCN2 as a biomarker for VaD. Therefore, we compared CSF LCN2 levels in different forms of dementia, VBI, and controls. Regarding the importance and known difficulties in the clinical differentiation between AD and VaD, we validated our results using independent cohorts. In addition, neuropathological analyses were performed to provide information on the location of LCN2 in brain tissue of patients with chronic multi-infarct dementia (MID), and those with AD.

Our results show that CSF LCN2 is elevated in patients with VaD compared to controls, cognitively unimpaired patients with VBI, and other forms of dementia. The high diagnostic accuracy highlights its potential as a biomarker for VaD in the differential diagnosis of dementia. Our neuropathological investigations display different expression of LCN2 in brains of patients with VaD and AD.

## Results

### Group descriptions

Data on age, sex and CSF biomarkers in all cohorts are presented in Table 1. In general, more samples from female patients were analysed (254 female, 218 male). VaD patients were older than those from other diagnostic groups. Patients with VaD, Lewy body dementias (LBD), and fronto-temporal dementia (FTD) showed similar profiles of established CSF biomarkers (t-tau, p-tau and amyloid beta 42). Patients with sporadic Creutzfeldt-Jakob disease (CJD) showed highly elevated CSF t-tau. Patients with AD and mixed dementia (MD, AD plus VaD) showed a typical signature of AD-pathology associated biomarkers^7^.

**Table 1:** Demographics and biomarkers data from the different cohorts.

	*n*	Age	Sex (f/m)	t-Tau (pg/mL)	p-Tau (pg/mL)	Amyloid β42 (pg/mL)	Lipocalin 2 (pg/mL)
*Cohort 1*							
ND	73	67 ± 10	40/33	232 ± 207	40 ± 11	719 ± 351	771 ± 347
AD	47	67 ± 10	26/21	591 ± 435	90 ± 51	449 ± 207	691 ± 247
VaD	27	72 ± 8	17/10	386 ± 445	44 ± 15	779 ± 348	2233 ± 1326
MD	31	74 ± 10	25/6	444 ± 272	76 ± 54	437 ± 206	1565 ± 833
LBD	36	69 ± 11	18/18	385 ± 301	56 ± 42	513 ± 292	917 ± 398
FTD	21	65 ± 12	8/13	373 ± 370	57 ± 21	665 ± 251	762 ± 331
CJD	54	68 ± 10	27/27	5936 ± 5914	61 ± 18	635 ± 324	1037 ± 661
SVDND	20	63 ± 14	9/11	181 ± 135	34 ± 16	787 ± 229	753 ± 300
VCIND	7	66 ± 9	2/5	177 ± 59	48 ± 12	1114 ± 220	946 ± 366
*Cohort 2*							
ND	24	64 ± 8	12/12	168 ± 74	35 ± 14	913 ± 409	713 ± 232
AD	15	68 ± 6	6/9	602 ± 336	71 ± 27	417 ± 95	660 ± 182
VaD	10	69 ± 10	3/7	330 ± 236	38 ± 13	768 ± 188	1440 ± 979
*Cohort 3*							
ND	15	67 ± 13	7/8	193 ± 57	33 ± 9	684 ± 123	753 ± 386
AD	27	72 ± 6	16/11	647 ± 307	88 ± 29	435 ± 81	709 ± 236
VaD	16	74 ± 6	10/6	402 ± 300	55 ± 23	501 ± 134	1381 ± 953
*Cohort 4*							
AD	28	72 ± 10	21/7	595 ± 234	86 ± 25	634 ± 228	634 ± 198
SVDND	3	71 ± 8	1/2	135 ± 37	27 ± 9	953 ± 55	613 ± 258
VCIND	8	67 ± 5	3/5	174 ± 45	38 ± 12	869 ± 328	845 ± 277
VaD	10	76 ± 6	3/7	248 ± 66	43 ± 11	932 ± 221	1131 ± 481

Number of cases (*n*), age (mean ± standard deviation), sex (female/male), CSF biomarkers total-Tau, p-Tau and amyloid beta 42 and CSF LCN2 (mean ± standard deviation in pg/mL) are indicated.

*ND* non-primarily neurodegenerative and non-ischemic neuropsychiatric diseases, *AD* Alzheimer’s disease, *VaD* vascular dementia, *MD* mixed dementia, *LBD* Lewy body dementia, *FTD* frontotemporal dementia, *CJD* sporadic Creutzfeldt-Jakob disease, *SVDND* small vessel disease no dementia, *VCIND* vascular cognitive impairment no dementia.

### LCN2 in the differential diagnosis of dementia

**Fig. 1:**
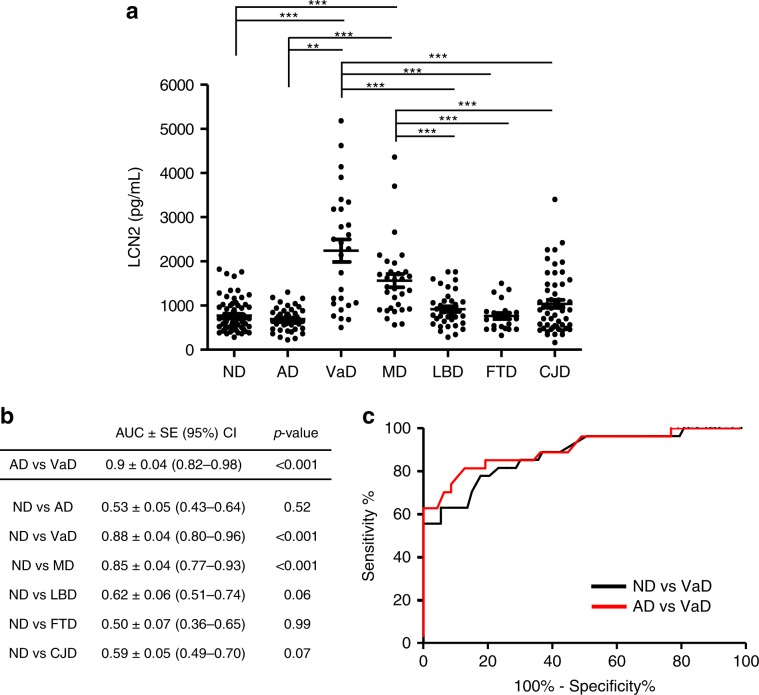
CSF LCN2 in the differential diagnosis of dementia (cohort 1). **a** LCN2 in non-primarily neurodegenerative and non-ischemic neuropsychiatric diseases (ND, *n* = 73), Alzheimer’s disease (AD, *n* = 47), vascular dementia (VaD, *n* = 27), mixed Alzheimer’s and vascular dementia (MD, *n* = 31), Lewy body dementias (LBD, *n* = 36), frontotemporal dementia (FTD, *n* = 21), and sporadic Creutzfeldt-Jakob disease (CJD, *n* = 54). Results are shown as mean ± SD for each condition. **b** Area under the curve (AUC) derived from receiver operating characteristic (ROC) curves, Standard Error (SE), 95% Confidence interval (95% CI) for LCN2 in the comparative analyses. **c** ROC curves of differentiating ND and VaD as well as AD and VaD. Differences between groups were analysed with Tukey contrasts using linear regression models controlled for age and sex. *p < 0.05, **p < 0.01, ***p < 0.001.

Cohort 1 included VaD (*n* = 27), MD (*n* = 31), AD (*n* = 47), LBD (*n* = 36), FTD (*n* = 21), CJD (*n* = 54), as well as non-primarily neurodegenerative and non-ischemic neuropsychiatric diseases (ND, *n* = 73). In a multi-comparative analysis corrected for covariates, LCN2 concentrations were significantly increased in VaD and MD compared to ND (*p* < 0.001, Tukey contrast for multiple comparisons of means) and other diagnostic groups (Table 1, Fig. 1a). No significant difference between VaD and MD could be observed. The diagnostic accuracy of LCN2 in the differentiation from ND is indicated by areas under the curve (AUC) and 95% confidence intervals (95% CI) in Fig. 1b, c. LCN2 concentrations discriminated ND from VaD (AUC = 0.88, 95% CI: 0.80–0.96, *p* < 0.001) and MD (AUC = 0.85, 95% CI: 0.77–0.93, *p* < 0.001) with high accuracy. LCN2 was able to discriminate AD from VaD (AUC = 0.9, 95% CI: 0.82–0.98, *p* < 0.001, *z* test with H_0_: AUC = 0.5 in all cases) with a sensitivity of 82% and a specificity of 87%, as well as ND from VaD with a sensitivity of 78% and a specificity of 82%. In contrast, LCN2 levels showed no diagnostic value in distinguishing AD, LBD, FTD, and CJD from ND (*p* > 0.05, *z* test with H_0_: AUC = 0.5).

### LCN2 discriminates VaD from AD in validation cohorts

**Fig. 2:**
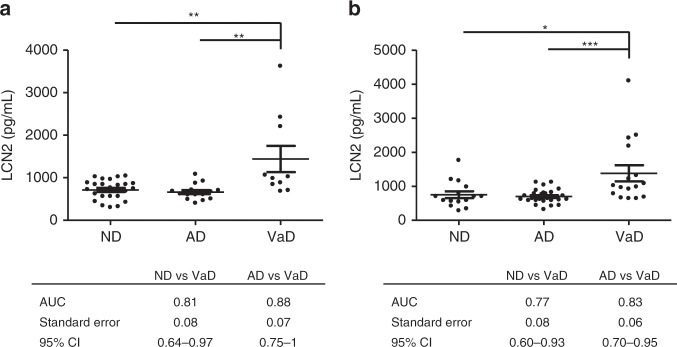
Diagnostic accuracy of CSF LCN2 in the discrimination of VaD and AD (cohorts 2 and 3). **a** Cohort 2: LCN2 concentrations in non-primarily neurodegenerative and non-ischemic neuropsychiatric diseases (ND, *n* = 24), Alzheimer’s disease (AD, *n* = 15), and vascular dementia (VaD, *n* = 10). Area under the curve (AUC) derived from receiver operating characteristic (ROC) curves, Standard Error, and 95% CI in the comparative analysis of VaD versus ND and AD. **b** Cohort 3: LCN2 concentrations in ND (n = 15), AD (n = 27), and VaD (n = 16). AUC derived from ROC curves, Standard Error, 95% CI in the comparative analysis of VaD versus ND and AD. Differences between groups were analysed with Tukey contrasts using linear regression models controlled for age and sex. *p < 0.05, **p < 0.01, ***p < 0.001.

**Fig. 3:**
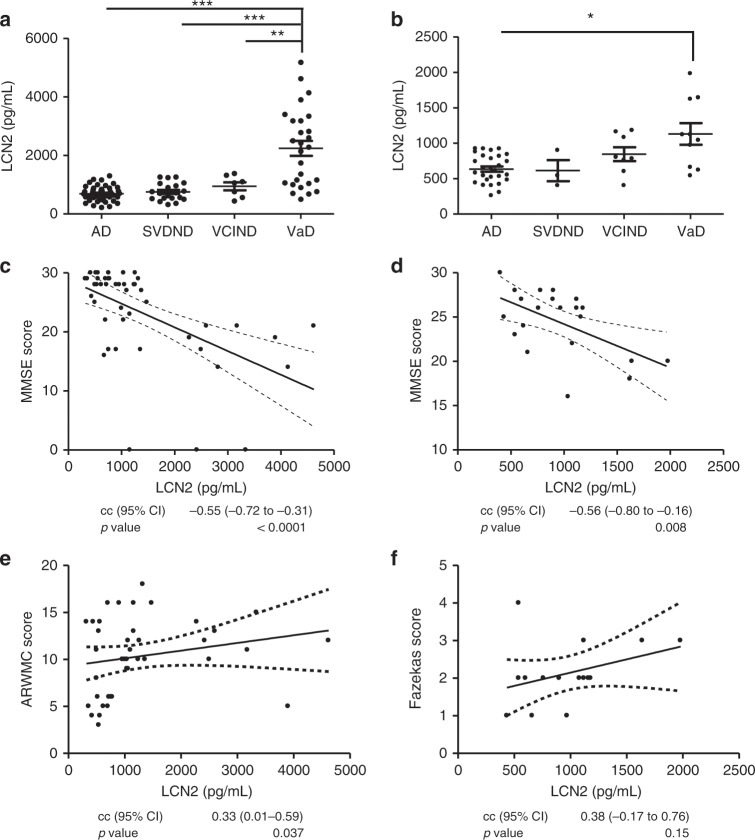
Associations of CSF LCN2, cognitive status, and white matter changes (cohorts 1 and 4). **a** Cohort 1: LCN2 levels in Alzheimer’s disease (AD, *n* = 47), small vessel disease no dementia (SVDND, *n* = 20), vascular cognitive impairment no dementia (VCIND, *n* = 7), and vascular dementia (VaD, *n* = 27). **b** Cohort 4: LCN2 levels in AD (*n* = 28), SVDND (*n* = 3), VCIND (*n* = 8), and VaD (*n* = 10). Differences between groups were analysed with Tukey contrasts using linear regression models controlled for age and sex. *p < 0.05, **p < 0.01, ***p < 0.001. **a**, **b** Mean ± SD is represented in the graphs. **c**, **d** Correlation analysis of LCN2 and Mini Mental Status Examination (MMSE) scores in SVDND, VCIND, and VaD in cohort 1 (*n* = 48) (**c**) and cohort 4 (*n* = 21) (**d**). Spearman correlation test, correlation coefficients (cc) and associated two-tailed *p* values. **e** CSF LCN2 and age-related white matter changes (ARWMC) in SVDND, VCIND, and VaD in cohort 1 (*n* = 50). Spearman correlation test, correlation coefficients (cc), and associated two-tailed *p* values. **f** LCN2 and Fazekas scale in SVDND, VCIND, and VaD in cohort 4 (*n* = 21). Spearman correlation test, correlation coefficients (cc), and associated two-tailed *p* values.

To validate the presence of elevated LCN2 concentrations in VaD, two independent cohorts (cohort 2 and 3) including ND, AD, and VaD cases were analysed with linear regression models adjusted for covariates and posterior multiple comparisons of means were performed with Tukey contrasts. In cohort 2, LCN2 was significantly increased in VaD (*n* = 10) compared to ND (*n* = 24, *p* < 0.01) and AD (*n* = 15, *p* < 0.01). It discriminated VaD from AD with an AUC of 0.88 (Fig. 2a). In cohort 3, LCN2 was significantly increased in VaD (*n* = 16) compared to ND (*n* = 15, *p* < 0.05) and AD (*n* = 27, *p* < 0.001). It discriminated VaD from AD with an AUC of 0.83 (Fig. 2b). AUCs derived from ND vs. VaD and AD vs. VaD comparisons were not significantly different (*z* test) between the cohorts (Supplementary Table 1). In cohort 4, LCN2 was significantly increased in VaD (*n* = 10) compared to AD (*n* = 28, *p* < 0.05, Tukey contrast) but no control group (ND) was available (Fig. 3b). In a separate statistical model including Mini Mental Status Examination (MMSE) score in the group of covariates, VaD groups showed significantly higher LCN2 levels than AD groups in all four cohorts, indicating that dementia stage does not significantly alter the results (Supplementary Table 2).

### VaD types, dementia stage, WMH, and albumin rati**o**

Regarding different types of VaD, LCN2 levels seemed to be similar in subcortical ischemic vascular dementia (SID) and multi-infarct (cortical) dementia (MID) while being lower in post-(single)stroke dementia (PSD) (Table 2). We could not investigate this observation further because case numbers were too low after building the diagnostic subgroups. Especially for PSD, only few cases were identified.

**Table 2:** Cognitive scores, white matter hyperintensities, and CSF LCN2 levels.

		*n*	MMSE (median score, min-max)	ARWMC (cohort 1) Fazekas (cohort 4) (median score, min-max)	Lipocalin 2 (mean pg/mL, SD)
*Cohort 1*					
AD		47	17 (0–27), *n* = 35	–	691 ± 247
VaD		27	19 (0–25), *n* = 21	12 (5–17), *n* = 23	2233 ± 1326
	SID	20	19 (0–25), *n* = 17	12 (5–16), *n* = 18	2232 ± 1340
	MID	5	17 (14–25), *n* = 3	12.5 (11–17), *n* = 4	2780 ± 1256
	PSD	2	16, *n* = 1	7, *n* = 1	870 ± 268
VCIND		7	28 (26–29)	12 (4–18)	946 ± 366
SVDND		20	28.5 (28–30)	6 (3–19)	753 ± 300
*Cohort 2*					
AD		15	20 (9–27), *n* = 14	–	660 ± 182
VaD^a^		10	13 (5–24), *n* = 9	NA	1440 ± 979
	SID	1	21	NA	840
	MID	6	16 (5–24)	NA	1853 ± 1092
	PSD	2	13 (13–13)	NA	970 ± 127
*Cohort 3*					
AD		27	21 (11–30)	–	709 ± 236
VaD^b^		16	23.5 (12–28)	NA	1381 ± 953
	SID	10	21.5 (14–28)	NA	1298 ± 1087
	MID	2	22 (20–24)	NA	2460 ± 56
*Cohort 4*					
AD		28	23 (15–28)	–	634 ± 198
VaD		10	21 (16–28)	2 (1–3)	1131 ± 481
	SID	7	26 (20–28)	2 (1–3)	1147 ± 512
	MID	3	18 (16–24)	1 (1–2)	1093 ± 502
VCIND		8	26 (22–28)	2 (1–2)	845 ± 277
SVDND		3	29 (28–30)	2 (2–4)	613 ± 258

^a^1 case data not available to determine type of VaD pathology.

^b^4 cases data not available to determine type of VaD pathology.

*n* number of cases, *AD* Alzheimer’s disease, *VaD* vascular dementia, *SID* subcortical ischemic vascular dementia, *MID* mulit-infarct cortical dementia, *PSD* post-(single)stroke dementia, *SVDND* small vessel disease no dementia, *VCIND* vascular cognitive impairment no dementia.

To assess the association between LCN2 and cognitive impairment in patients with cerebrovascular pathology, LCN2 concentrations were quantified in two cohorts that included cerebral small vessel disease but no dementia (SVDND), vascular cognitive impairment but no dementia (VCIND), and VaD (cohort 1 and 4). AD cases were included in the multi-comparative analysis (linear regression adjusted for covariates) in order to compare baseline LCN2 in non-vascular pathology. In cohort 1, LCN2 concentrations in VaD (*n* = 27) were significantly different from AD (*n* = 47), SVDND (*n* = 20), and VCIND (*n* = 7) (*p* < 0.01, Tukey contrast for multiple comparisons of means) (Fig. 3a). In cohort 4, VaD (*n* = 10) displayed higher LCN2 concentrations than AD (*n* = 28), SVDND (*n* = 3), and VCIND (*n* = 8), although significant differences were only detected between AD and VaD groups (*p* < 0.05) (Supplementary Table 1, Fig. 3b). Additional association analyses of LCN2 and MMSE scores including all groups with cerebrovascular disease (SVDND, VCIND, and VaD) showed highly significant negative correlations in cohort 1 (cc = −0.55, *p* < 0.0001, Fig. 3c, Spearman correlation test) and cohort 4 (cc = −0.56, *p* = 0.008, Fig. 3d, Spearman correlation test). In AD groups, we observed no significant correlation (cohort 1: cc = 0.16, cohort 4: cc = −0.06, Supplementary Fig. 1a, Supplementary Fig. 1b).

We investigated the association of white matter changes and LCN2 in patients with cerebrovascular disease (SVDND, VCIND, and VaD) in cohorts 1 and 4 excluding patients with radiological evidence for cortical infarctions (Fig. 3e, f). LCN2 levels showed a significant positive correlation with the Age-Related White Matter Changes (ARWMC) scale in cohort 1 (cc = 0.33, *p* = 0.037, Spearman correlation test) and a non-significant positive correlation with the Fazekas scale in cohort 4 (cc = 0.38, *p* = 0.15, Spearman correlation test).

The CSF/serum albumin ratio (QAlb) is a well-known marker of blood-brain-barrier (BBB) function and was measured in the continuum of vascular pathology in cohort 1 and 4. It was significantly elevated in VaD compared to SVDND (*p* < 0.05 One-way ANOVA Bonferroni’s post hoc) (Supplementary Fig. 2a), presenting a strong positive correlation with LCN2 in SVDND and VaD and non-significant positive correlation in VCIND (Supplementary Fig. 2b).

### Association with demographics and other biomarkers

LCN2 showed a weak non-significant positive correlation with age in all cohorts when all diagnostic groups were analysed (Supplementary Table 3). Yet, all group comparisons were adjusted for age and sex (Fig. 1, Fig. 2, Supplementary Fig. 2, Supplementary Table 2) to exclude that these factors behave as relevant confounders. Associations between LCN2 concentrations and demographic parameters as well as CSF total-Tau, p-Tau, and amyloid beta 42 were analysed in all VaD (*n* = 62) and AD (*n* = 117) cases. In VaD, LCN2 showed a significant positive correlation with total-Tau (cc = 0.34, *p* = 0.006, Spearman correlation test) but not with other studied parameters (Table 3). In contrast, no significance was observed in AD (cc = 0.05, *p* = 0.57, Spearman correlation test). Since LCN2 has been discussed as a blood-based marker for kidney injury, we retrospectively reviewed medical files of all AD and VBI cases to identify those with kidney injury and included this condition in the regression model as an additional covariate. It was not a significant variable in the model (*p* = 0.711 for the significance of the β estimate) and differences of LCN2 levels between groups were not affected. Therefore, we did not consider the presence of kidney injury to be a significant confounder.

**Table 3:** Correlations between CSF LCN2 levels, demographics, and CSF biomarkers.

	VaD			AD		
	*n*	cc	*p* value	*n*	cc	*p* value
*Demographics*						
Age	63	0.11	0.37	117	0.19	0.12
Sex	63	–	0.90	117	–	0.15
*CSF biomarkers*						
t-Tau	63	0.34	0.006	117	0.05	0.57
p-Tau	63	0.05	0.69	117	−0.12	0.20
Amyloid β42	63	−0.10	0.43	117	−0.06	0.48

Age, sex and CSF biomarkers (total-Tau, p-Tau and amyloid beta 42) in vascular dementia (VaD) and Alzheimer’s Disease (AD) cases from all cohorts were tested for normality and Spearman (LCN2 vs biomarkers), Pearson (LCN2 vs age) correlation tests and Mann–Whitney test (LCN2 vs sex) were applied. Correlation coefficients (cc) and associated *p* values are reported. *N*: number of cases.

### LCN2 expression in the brain of AD and VaD

**Fig. 4:**
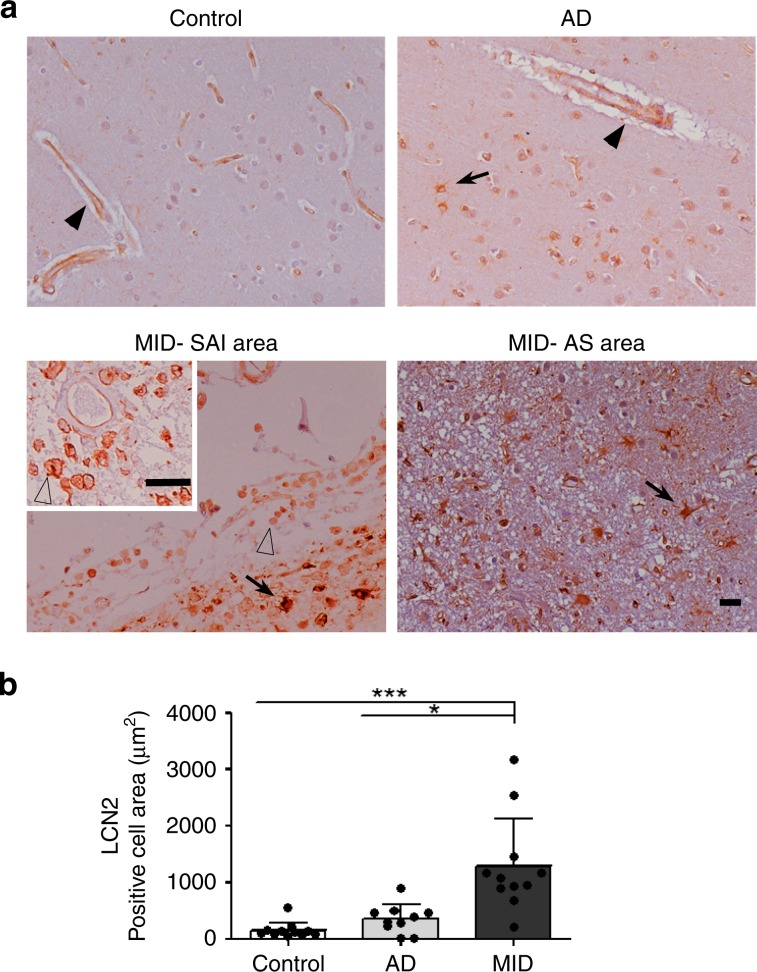
LCN2 expression in control, AD, and MID brain tissue. **a** LCN2 immunohistochemistry in the cerebral cortex of control, Alzheimer’s disease (AD), and Multi-Infarct Chronic Encephalopathy (MID) cases, including subacute infarct areas (MID-SAI area) and astrocytic scar areas (MID-AS area). LCN2 staining is observed in intact blood vessels in controls, AD and MID cases (arrow-heads). Increased LCN2 expression is observed in reactive astrocytes in AD and MID (arrows) and in monocyte/macrophage cells of the MID-SAI area (empty arrow-heads). Paraffin sections counterstained with hematoxylin. Bar: 25 µm; insert-bar 50 µm. **b** Quantification of LCN2 positive cells area in cerebral cortex and striatum in µm^2^. Significant increase of LCN2 positive cells area in MID cases with respect to controls (***p < 0.001) and AD cases (*p < 0.05). Shown results are means (±SD) of controls (*n* = 11), AD (*n* = 10), and MID (*n* = 11) cases. In MID cases, area of subacute infraction (n = 6) and chronic infarcts (*n* = 11) were analysed. Results were analysed by Kruskal-Wallis followed by Dunn's Multiple Comparison test.

We included controls (*n* = 11, mean age ± SD, 56.3 ± 8.4), “pure” AD (co-morbidities were restricted to minimal small vessel disease, *n* = 10, mean age ± SD: 76.6 ± 6.0), and MID cases (*n* = 11, mean age ± SD: 72.5 ± 11.4) for neuropathological studies. In control brains, we detected LCN2 immunoreactivity in capillaries and resting microglia (Fig. 4a, upper- left). In AD cases (stages V and VI), LCN2 immunoreactivity was preserved in capillaries but increased in a subpopulation of astrocytes localized around β-amyloid plaques or layered in the cerebral cortex, and in reactive microglia (Fig. 4a, upper-right). Lesions at different evolution states were observed in all cases with MID. These lesions included rare acute infarcts in which acute necrosis was the predominant alteration, subacute infarcts with abundant macrophages and variable peripheral reactive astrocytes, and old, often cystic, infarcts with a necrotic center surrounded by a scar of astrocytes (chronic reactive astrocytosis). Massive LCN2 immunoreactivity was observed in subacute infarct areas (MID-SAI area) and in macrophages (Fig. 4a, bottom- left insert). Additional LCN2 immunoreactivity occurred in reactive astrocytes as seen in small numbers in the peripheral region of subacute infarcts (Fig. 4a, bottom left) and in the astrocytic scar area surrounding a cystic area in large infarcts or replacing deceased neurons in small infarcts (MID-AS area) (Fig. 4a bottom right). Total LNC2 staining was analysed by Kruskal-Wallis followed by Dunn's Multiple Comparison test showing an increased immunoreactivity in MID in comparison with AD (*p* < 0.05) and controls (*p* < 0.001, Fig. 4b).

**Fig. 5:**
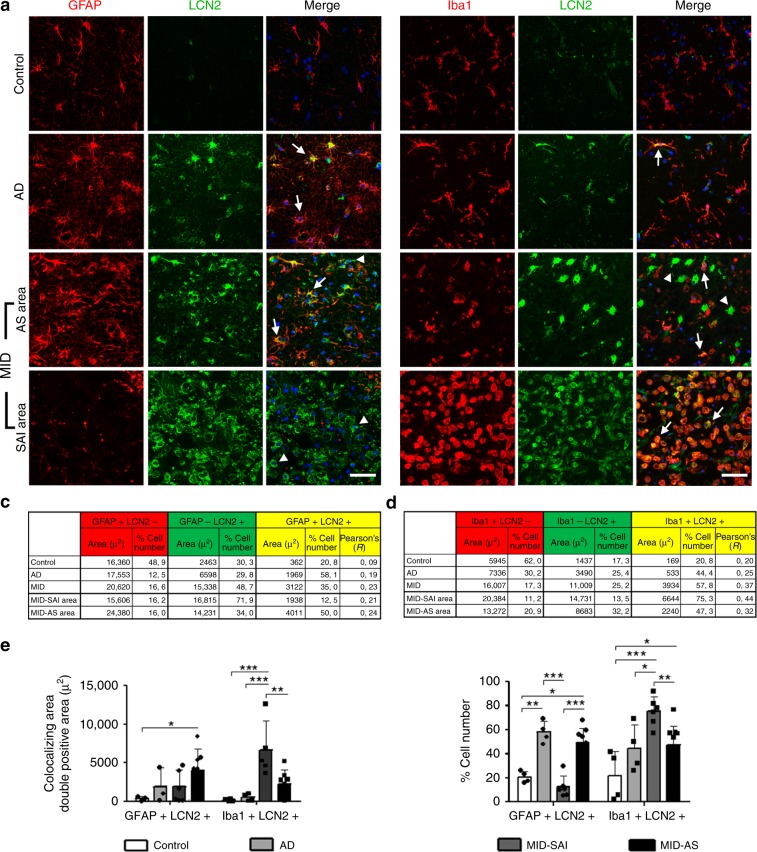
LCN2 expression in brain tissue and association with glial markers. **a** LCN2 and GFAP double-immunostaining in the cerebral cortex of control, AD, and MID cases. LCN2 co-localises with GFAP immunoreactive astrocytes (arrows) in AD, with surrounding plaque like structures, and in MID in astrocytic scar area (MID-AS). Abundant LCN2+GFAP- cells are observed in MID at the subacute infarction area (MID-SAI; arrow-heads), where there is little astrocyte presence. Bar = 50 µm. **b** LCN2 and Iba1 double-immunostaining in the cerebral cortex of control, AD, and MID cases. LCN2 co-localises with Iba1 immunoreactive microglia in AD and MID-AS (middle panels, arrows). In MID-SAI, predominant Iba1 positive staining was observed, displaying almost entire colocalisation with LCN2+ cells (bottom panel, arrows). LCN2+Iba1- cells are observed in MID-AS slices (arrow-heads). Bar = 50 µm. **c**, **d** Quantification of (**c**) LCN2 and GFAP, and (**d**) LCN2 and Iba1 double-immunostainings. Tables show single- and double-stained area (µm^2^), percentage of cells and Pearson’s colocalisation coefficient in Control, AD, and MID (total and divided in MID-SAI and MID-AS). **e** Graphic representation of double-stained area (left panel) and percentage of double-stained cells (right panel) in control, AD, MID-SAI, and MID-AS. Data are shown as mean ± SD of control (*n* = 4), AD (*n* = 4), and MID (*n* = 10). MID-AS areas were quantified in 10 cases and MID-SAI areas in 6 cases. Mean ± SD is included for each graph. Two-way ANOVA followed by Bonferroni’s post hoc test were used to analyse results *p < 0.05, **p < 0.01, ***p < 0.001.

Double-labeling immunofluorescence to GFAP and LCN2 showed that about 58% of astrocytes in AD, 12% of astrocytes in the peripheral region of subacute infarcts, and 32% of astrocytes at the periphery of chronic infarcts expressed LCN2 (Fig. 5a, c, e). In contrast, double-labeling immunofluorescence disclosed that about 75% of Iba1-positive cells in subacute infarcts expressed LCN2. About the same percentage or even more LCN2-immunoreactive cells were macrophages (Fig. 5b, d, e).

IgG and Fibrinogen immunoreactivity was detected in the perivascular space in AD and VaD, but not in controls (Supplementary Fig. 3), suggesting BBB impairment in both diseases and the absence of differential contribution of BBB alteration to the CSF concentrations of LCN2 in AD and VaD.

## Discussion

Imaging markers like N-acetyl aspartate (NAA) in proton magnetic resonance spectroscopy for Binswanger’s disease^29^ and candidate CSF markers like Qalb^30,31^, neurofilament light^32^, and matrix metalloproteinases^33^ for VaD have been suggested in recent years. These markers have shown uncertain or at best moderate diagnostic accuracy^34,35^.

The results from cohort 1 show that LCN2 levels might allow accurate differentiation between VaD and neurological controls as well as all other investigated causes of dementia. The broad spectrum of different dementia types analysed underlines the potential clinical value of the marker. However, cases numbers were rather low. Therefore, we investigated two independent cohorts (cohort 2 and 3) and were able to validate our findings through multi-national European cooperation. The results represent a very robust proof of concept.

Clinical and MRI data from cohort 1 and 4 suggest that higher LCN2 levels are associated with the degree of cognitive impairment in VBI, but not in AD. Regarding the different types of VaD, LCN2 is possibly lower in patients with PSD, but low case numbers precluded validating this observation statistically. Only in cohort 1, the correlation of high LCN2 levels and WMH load showed (borderline) significance. All this may indicate that LCN2 is closely associated with clinical disease stage in VaD rather than with common imaging markers. Further investigations and deeper analyses of MRI markers (atrophy, infarct volume, WMH volume, etc.) will need to explore these assumptions and the presence of VaD patients with unaltered or only slightly elevated CSF LCN2 levels in all cohorts. Here, we focused on the association between LCN2 levels and clinical diagnosis.

A previous study showed elevation of plasma LCN2 in patients with amnestic MCI but, in line with our results, not in demented patients with AD^23^. Inflammatory processes in early AD stages were suggested as a possible cause but concomitant vascular pathology was not excluded and diagnostic accuracy was not evaluated. Another study reported an elevation of CSF LCN2 and a positive correlation with neurofilament light in patients with progressive multiple sclerosis, but the increase was moderate and the authors stated that LCN2 was likely not a suitable biomarker for the clinical diagnosis of this condition^24^.

Considering this, LCN2 may be a valuable biomarker for VaD in the context of differential diagnosis. Such a marker has long been pursued^17,18,34^. Besides new imaging markers that display functional consequences of vascular lesions^29,36^, fluid markers like LCN2 are candidates to contribute to a biomarker-based definition of VaD as has recently been proposed for AD^7^.

LCN2 is synthesized and secreted as an inducible factor from activated microglia, reactive astrocytes, neurons, and endothelial cells in response to inflammatory, infectious, and injurious insults, in all of which it plays varied functions^37^. Several lines of evidence implicate LCN2 in the progression of cerebral infarcts^38^.

Because the pathophysiology of LCN2 in VaD has not yet been resolved, caution is necessary when disclosing the molecular causes of LCN2 alterations in biological fluids. In cohort 1 and cohort 4, Qalb was strongly associated with LCN2 levels. Since disturbance of the BBB is a core feature of the pathophysiology of VaD^39^, elevated CSF LCN2 might be related to impaired BBB function like it has been assumed to be the case for Qalb. However, LCN2 seems to differentiate VaD from other forms of dementia better than what has been shown for Qalb^30,31,34^. Furthermore, LCN2 also displays significant positive correlation with CSF total-Tau in VaD but not in AD (Table 3), which might indicate an association with the extent of neuronal damage.

Neuropathological examination of control brains indicates that LCN2 is expressed in capillaries and microglia, but rarely in astrocytes. In AD brains, LCN2 is expressed in reactive microglia and reactive astrocytes. In MID brains, LCN2 is expressed in macrophages located in subacute infarcts, in reactive astrocytes at the periphery of subacute infarcts, and in chronic infarcts. Previous studies associated LCN2 with inflammation and cellular damage in cerebral inflammatory diseases such as multiple sclerosis and neuropsychiatric lupus^24,40,41^. LCN2 is involved in the inflammatory activation of astrocytes^42^. Hypoxia induces astrocyte-derived LCN2 in ischemic stroke^43^ while astrocyte-derived LCN2 mediates hippocampal damage and cognitive deficits in experimental models of VaD^28^.

It is tempting to speculate that high LCN2 levels in the brain may be expected in conditions with robust astrocytic gliosis such as CJD. However, CSF LCN2 in CJD was not significantly altered in multi-comparative analyses. Therefore, other scenarios may be considered. VaD may present with a particular profile of reactive astrocytes. Microglia and macrophages may be additional sources of LCN2, and areas of subacute infarcts in MID are particularly rich in macrophages. Unfortunately, comprehensive studies combining clinical data, high sensitivity MRI, and CSF LCN2 levels in the progression of ischemic infarcts are not available. In addition to alterations of the BBB, cerebrovascular diseases, and particularly MID in the present context, show disruption of the interface between blood vessels and Virchow-Robin spaces as well as between Virchow-Robin spaces and brain tissue, which adjoin to the subventricular space and the CSF. This represents another considerable mechanism regarding abnormal protein extravasation of secreted brain proteins to the CSF.

Our study concerns an important area of research with major socioeconomic relevance. The participating centres are specialized institutions capable of defining patient groups with great expertise in applying clinical and para-clinical assessments. The multi-centre setting also allowed for demonstration of the reproducibility of our findings.

The absence of a reliable gold standard for the clinical diagnosis of VaD is a limitation of our study. The reference standard^2^ and its key markers probably lack high accuracy^3,11,12^. We assume that the inclusion of independent cohorts with independent diagnostic evaluations is an appropriate way to regard this problem and to validate the clinical reliability of the results. The frequent occurrence of concomitant pathologies (VaD and AD) is a problem that we tried to address by differentiating (cohort 1) or excluding (cohorts 2, 3, 4) patients with clinical evidence of possible MD. A biomarker for “pure” VaD may also be useful in the differential diagnosis of MD when used in combination with AD-related biomarkers. Since these biomarkers were part of the reference standard for the diagnosis of AD, we could investigate this further. In general, clinical evaluations of biomarkers of VaD have to be interpreted with caution.

The retrospective study design and low case numbers in each cohort have several limitations. We could not investigate relations between kidney disease (for which LCN2 is considered to be a promising biomarker), peripheral LCN2, and CSF LCN2 further. Thus, we cannot be sure that elevated CSF LCN2 is brain-derived and we cannot state a specific causal relationship between LCN2 and VBI.

No sample-size calculation was performed. Different forms of VaD^8,44^ were stratified but not statistically analysed. Advanced neuropathological differentiation of cases was not available for the clinical cohorts and it cannot be ruled out that other subtypes of VaD (e.g. multi-infarct encephalopathy vs. sole diffuse alteration of the white matter) may account for differences of CSF LCN2 levels within the VaD groups. However, this did not result in low overall diagnostic accuracy. We performed basic correlation analyses of LCN2 and MRI markers as well as neuropathological investigation of certain VaD subtypes, but detailed data will have to be acquired through prospective studies to validate the findings.

Due to the case-control design, the cohorts may be biased. Especially in cohort 1, many samples were collected in the framework of differential diagnosis of atypical or rapidly progressive dementia, which may explain higher LCN2 levels compared to the other cohorts. Non-identic population characteristics and pre-analytical conditions in the study centres cannot be ruled out. By contrast, the design and the heterogeneity of cohorts is also a strength of the study. We could show that different LCN2 levels in AD and VaD were consistent even though diagnostic procedures and case selection were not performed in the same centre.

The presented clinical data indicate that CSF LCN2 is a marker for dementia associated with VBI and that it has the potential to become a complementary tool in the differential diagnosis of VaD and AD. Since this is the first clinical evaluation of LCN2 in this context, further investigation is needed before it can be included in the clinical work-up. Our findings set the hallmark for further neuropathological and prospective clinical investigations.

## Methods

### Study design and study centres

CSF analyses had been planned in Göttingen before 472 samples were collected from four European centres in the framework of a retrospective case-control study design. The case selection was based on the availability of samples from patients with the target conditions (VaD and important differential diagnoses) and complete information for clinical criteria application (including basic CSF analyses and neuroimaging). Patients with central nervous system inflammation and neoplasia (possible causes for white matter pathology) as well as intermediate diagnostic categories (e.g. possible AD, CJD, DLB etc.) were excluded. CSF LCN2 analyses (index test) were performed in Göttingen blind to diagnoses in 2018.

Cohort 1 was recruited at the Clinical Dementia Center and the National Reference Center for Creutzfeldt-Jakob disease surveillance at the University Medical Center of Göttingen (Germany) and included ND, VaD, AD and other neurodegenerative dementias as well as patients with VCIND and SVDND. Cohort 2 (ND, AD, and VaD) was recruited at the Dementia Clinic, Neurology Department of Coimbra-University Hospital (Portugal). Cohort 3 (ND, AD, and VaD) was recruited at the Geriatric Clinic, Linköping University Hospital, Linköping (Sweden). Cohort 4 (AD, VaD, VCIND, and SVDND) was recruited at the Center of Cognitive Neurology and Inserm, Lariboisière Hospital, University Paris Diderot (France). Case numbers are shown in Table 1.

### Diagnostic criteria

The clinical classification of patients and assessment of imaging data was performed in their respective study centres by specialized neurologists and neuroradiologists. Probable AD was diagnosed according to the National Institute on Aging—Alzheimer's Association workgroups (NIA-AA) criteria^45^ (cohort 1,2 and 4) and National Institute of Neurological and Communicative Disorders and Stroke and the AD and Related Disorders Association (NINCDS-ADRDA) criteria^46^ (cohort 3). Patients with coexisting pathology (AD plus VBI) were excluded from the AD group as much as possible. Only patients without significant vascular brain lesions (on MRI, rated by the local neuroradiologists) and no specific clinical signs for cerebrovascular disease (e.g. strokes, stroke-like episodes, stepwise worsening of symptoms, etc.) were included.

VaD diagnosis in all four centres was based on clinical and radiological criteria described by the National Institute of Neurological and Communicative Disorders and Stroke and the AD and Related Disorders Association (NINDS-AIREN^2^). VaD diagnosis also included a complete clinical work up showing no evidence for other than vascular pathology of the brain.

ND patients were diagnosed according to acknowledged standard neurologic clinical and para-clinical findings based on the International Classification of Diseases (ICD) 10 definitions. On MRI or CT, these patients did not show any cerebrovascular lesions other than normal age-related WMH (as rated by neuroradiologists during the diagnostic process). Neuroimaging and lumbar puncture were performed for various reasons (e.g. headaches, affective disorders etc.). ND patients did not show any clinical signs of cognitive impairment but a detailed neuropsychological test battery had not been performed in many of them.

Data from patients with MD, LBD, FTD, and CJD was only available in cohort 1. The MD group included patients according to clinical International Working Group (IWG-2) criteria^47^ and also patients with VaD according to NINDS-AIREN criteria plus at least one AD-typical CSF biomarker (elevated phosphorylated-tau or low amyloid beta 1-42/1-40 ratio). The LBD group included dementia with Lewy bodies^48^ and Parkinson’s disease dementia^49^. The FTD group included only behavioral variant FTD (bvFTD) and was diagnosed according to the International Behavioral Variant FTD Criteria Consortium for bvFTD^50^. Patients with Sporadic Creutzfeldt-Jakob disease (CJD) were classified as probable or definite according to WHO criteria^51^.

### Classification of cerebrovascular disease

All clinical cohorts included different forms of VaD while for morphological studies and immunohistochemistry, only brains with chronic multi-infarct encephalopathy were available.

We analysed clinical and imaging data from all VaD patients to assign disease type (PSD, SID, and MID) according to the Vascular Impairment of Cognition Classification Consensus Study^8^. SID includes cases with severe damage of the cerebral white matter often accompanied by status cribosus and lacunar infarcts, MID is characterized by multiple infarcts involving the cerebral cortex but also other parts of the brain, PSD refers to cases of dementia resulting from a single stroke.

Cohort 1 and cohort 4 also inlcuded patients with SVDND and VCIND. Patients with VCIND showed cognitive impairment but no significant impairment of activities of daily living. Neuropsychological assessment (Cambridge Cognitive Examination battery in cohort 1 and Consortium to Establish a Registry for AD battery in cohort 4) did not reveal reduced total or subscale scores in patients with SVDND (>−1.5 SD, matched for sex, age, and education). The diagnosis of subcortical small vessel disease was based on MRI (FLAIR or T2 weighted images) showing ≥4 points on the ARWMC^52^ scale in cohort 1 and either rank 3 on the Fazekas scale or multiple lacunae in cohort 4. Patients received MRI for various reasons (e.g. headaches, affective disorders etc.).

### CSF analyses

CSF LCN2 was quantified using the human LCN2/NGAL (Neutrophil Gelatinase-Associated Lipocalin) Quantikine Enzyme-linked Immunosorbent Assay (ELISA) Kit from R&D according to the manufacturer’s instructions (R&D Systems, Inc. Minneapolis, MN). CSF samples were diluted 1:2. Inter- and intra-assay coefficients of variation were below 12 and 10%, respectively. The limit of quantification was 0.052 (52 pg/ml) and the limit of detection was 0.023 (23 pg/ml). Total-Tau, phospho-Tau (p-Tau), and amyloid beta 42 were quantitatively measured using ELISA kits from Fujirebio (Fujirebio, Ghent, Belgium). QAlb (CSF albumin/serum albumin*10^3^) was measured by local neurochemistry laboratories (cohort 1 and cohort 4) according to standard methodology. Test performers were blind to clinical information and clinical investigators vice versa.

### Morphological studies and immunohistochemistry

Morphological studies were carried out in post mortem human brains obtained from the Institute of Neuropathology brain bank, HUB-ICO-IDIBELL Biobank. One hemisphere was immediately cut in coronal sections, 1 cm thick. Selected areas of the encephalon were rapidly dissected, frozen on metal plates over dry ice, placed in individual air-tight plastic bags, and stored at −80 °C until use for biochemical studies. The other hemisphere was fixed by immersion in 4% buffered formalin for morphological studies; sections from representative regions were stained with hematoxylin/eosin, periodic acid-Schiff and Klüver-Barrera, or processed for immunohistochemistry analysis. Cases were categorized as controls (cerebral cortex frontal area 8 and striatum, *n* = 11), AD (cerebral cortex frontal area 8, AD stage V-VI/C, *n* = 10), and MID (cerebral cortex, temporal cortex, striatum, including areas with acute, subacute, and chronic infarcts, *n* = 11). Control cases had not suffered from neurologic or psychiatric diseases, infections of the nervous system, brain neoplasms, or systemic and central immune diseases, and did not have abnormalities in the neuropathological examination. Mixed pathologies were excluded and cases with circulatory/vascular-linked diffuse white matter encephalopathy and cases with PSD were not included in the study. CSF was not available in any of the studied post-mortem brain series.

De-waxed sections, 4-μm thick, were processed for immunohistochemistry. The sections were boiled in citrate buffer (20 min) to retrieve antigenicity. Endogenous peroxidases were blocked by incubation in Dako Real Peroxidase blocking solution (Dako, S2023). Then, sections were incubated at 4 °C overnight with one of the primary antibodies properly diluted in Dako Real Antibody Diluent (Dako, S2022) and afterwards incubated with MultiLink biotinylated antibody followed by HRP-conjugated streptavidin (BioGenex QP9009L-E). The peroxidase reaction was visualized with diaminobenzidine (DAB; Sigma-Aldrich, D5637) and H_2_O_2_. Control of the immunostaining included omission of the primary antibody. Nucleus labeling was obtained by Hematoxylin staining. For immunofluorescence, 4-μm-thick de-waxed sections were used. After antigen retrieval, slices were incubated in Sudan Black for 15 min to reduce lipofuscin autofluorescence. Unspecific bonding was blocked by Fetal Bovine Serum (FBS) 10% 1h; afterwards sections were incubated at 4 °C overnight with one of the primary antibodies properly diluted in FBS 10%. Appropriate Alexa-fluor 488 and 555 were used as secondary antibodies. Nucleus labeling was performed using DAPI. LCN2 antibody (RD Systems MAB1757) was used at 1:50 dilution, GFAP antibody (Dako Z0334) was used at 1:400 dilution, Iba1 antibody (Wako 019-19741) was used at 1:1000 dilution, IgG heavy chain antibody (Proteintech 16402-1-AP) was used at 1:200 dilution, and Fibrinogen FGL2 antibody (Proteintech 11827-1-AP) was used at 1:100 dilution.

### LCN2 quantification in brain tissue

Immunohistochemistry and immunofluorescence pictures were taken with a Nikon Eclipse E-800 microscope and ProgRes Capture Pro 2.7.7 software. LCN2 immunostaining was quantified in 11 controls, 10 AD (stage V-VI/C), and 11 MID cases. Ten images of each slice were taken throughout the tissue, including areas of subacute and chronic infarcts in MID slices. Pictures were analysed using Fiji ImageJ software; LCN2 positive area was measured in µm^2^ only in particles >25 µm^2^ by stablishing a fixed positive staining threshold.

For immunofluorescence, 4 controls, 4 AD, and 10 MID cases were tested. From MID, six slices were quantified in subacute infarctions (MID-SAI) and 10 in the astrocytic scar in chronic infarcts (MID-AS). Six pictures of each slice were analysed using Fiji ImageJ software. Three different methods were applied. The first one analysed number of green, red and yellow pixel areas using colocalization thresholded plugin. The second one used Coloc2 plugin to obtain Pearson’s colocalization coefficients of all pictures. In addition, two independent researchers counted the number of green, red and yellow cells manually in a double blind study.

### Statistical analysis

Group differences of LCN2 levels were assessed through linear regression analyses. We log-transformed LCN2 concentration and built models adjusting for age and sex as potential confounders. Multiple comparisons among diagnostic groups were made with multcomp package in R^53^. We performed additional comparisons of LCN2 levels between AD and VaD in the four cohorts, controlling for dementia stage through models that include age, sex, and MMSE score as covariates. Models including kidney disease in the group of covariates were built in order to analyse the potential role of kidney injury as a confounder of LCN2 levels.

Spearman rank correlation coefficients were used to assess associations between continuous biomarker levels, ARWMC, and MMSE scores. LCN2 level association with demographic data was investigated using Pearson correlation (age) or Mann–Whitney test (sex).

To determine biomarker diagnostic accuracy, ROC curve analyses were carried out and AUC with 95% CI were calculated. Values of sensitivity and specificity were derived from best cut-off points in cohort 1 according to Youden’s index. Comparisons between AUC values were performed with DeLong’s test available in pROC R package^54^.

For group comparisons of immunohistochemistry and immunofluorescence results, Kruskal-Wallis followed by Dunn’s Multiple Comparison test and two way-ANOVA followed by Bonferroni’s post hoc test were applied respectively, using GraphPad Prismv5 software. In all analyses, statistical significance was considered at *p* < 0.05.

### Ethics

The study was conducted according to the revised Declaration of Helsinki and Good Clinical Practice guidelines. Informed written consent was obtained from participants and/or their relatives. The study of CSF samples and case data was approved by the ethics committees of the University Medical Center Göttingen (Germany), Linköpping (Sweden), Paris University Hospitals (France), and the Ethics Board of Coimbra University Hospital (Portugal).

Post mortem brain tissue was obtained from the Institute of Neuropathology brain bank, HUB-ICO-IDIBELL biobank following the guidelines of Spanish legislation (Real Decreto de Biobancos 1716/2011).

### Reporting summary

Further information on research design is available in the Nature Research Reporting Summary linked to this article.

## Supplementary information

Supplementary information

Reporting Summary

## Data Availability

The datasets generated during and/or analysed during the current study contain patient-related clinical information and are not publicly available. However, anonymised raw data to generate figures and tables are available from the corresponding authors on reasonable request.
