# Supplementary material for: Cerebrospinal fluid lipocalin 2 as a novel biomarker for the differential diagnosis of vascular dementia
Source: Nat Commun. 2020 Jan 30;11:619. doi: 10.1038/s41467-020-14373-2 (PMC6992814; doi:10.1038/s41467-020-14373-2)
Supplement: Supplementary file 1 — Supplementary information [file 41467_2020_14373_MOESM1_ESM.pdf]

Supplementary information

Cerebrospinal fluid lipocalin 2 as a novel biomarker for the differential diagnosis of vascular dementia

Authors

Llorens F*°, Hermann P*°, Villar-Piqué A*, et al.

*equal contribution

°corresponding authors

Correspondence can be addressed to:

Franc Llorens, PhD: franc.llorens@gmail.com

Peter Hermann, MD: peter.hermann@med.uni-goettingen.de

Supplementary Figure 1.

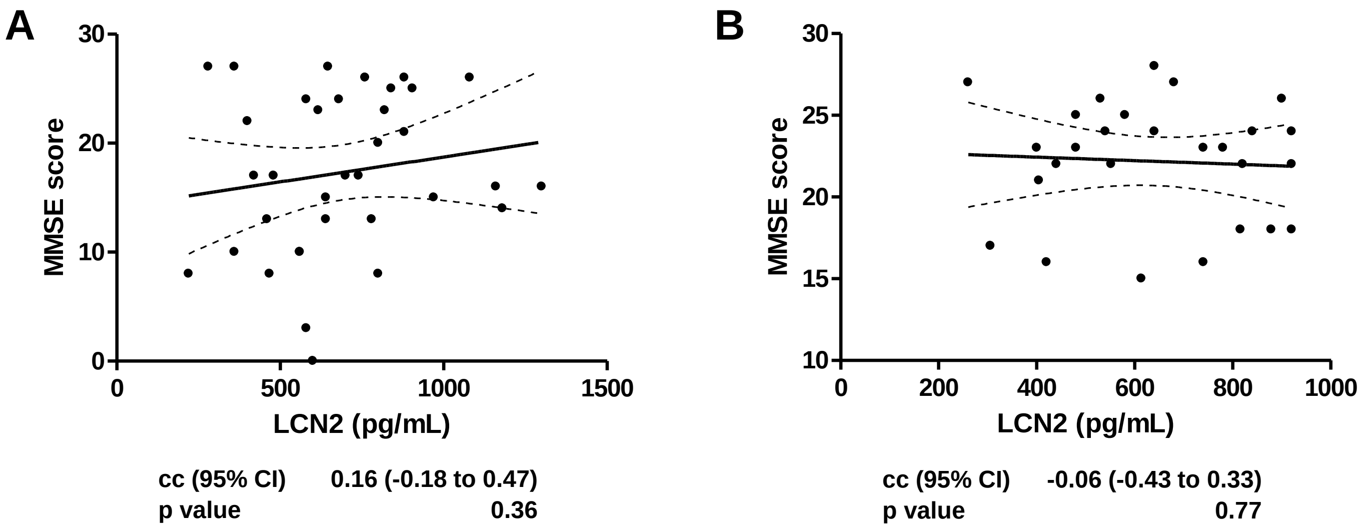

Association of CSF LCN2 and MMSE scores in AD groups.

(A) LCN2 and mini mental status examination (MMSE) scores in Alzheimer's disease (AD), cohort 1 (n=35). (B) LCN2 and MMSE scores in AD, cohort 4 (n=28). Spearman correlation test, correlation coefficients (cc), and associated two-tailed p values.

Supplementary Figure 2.

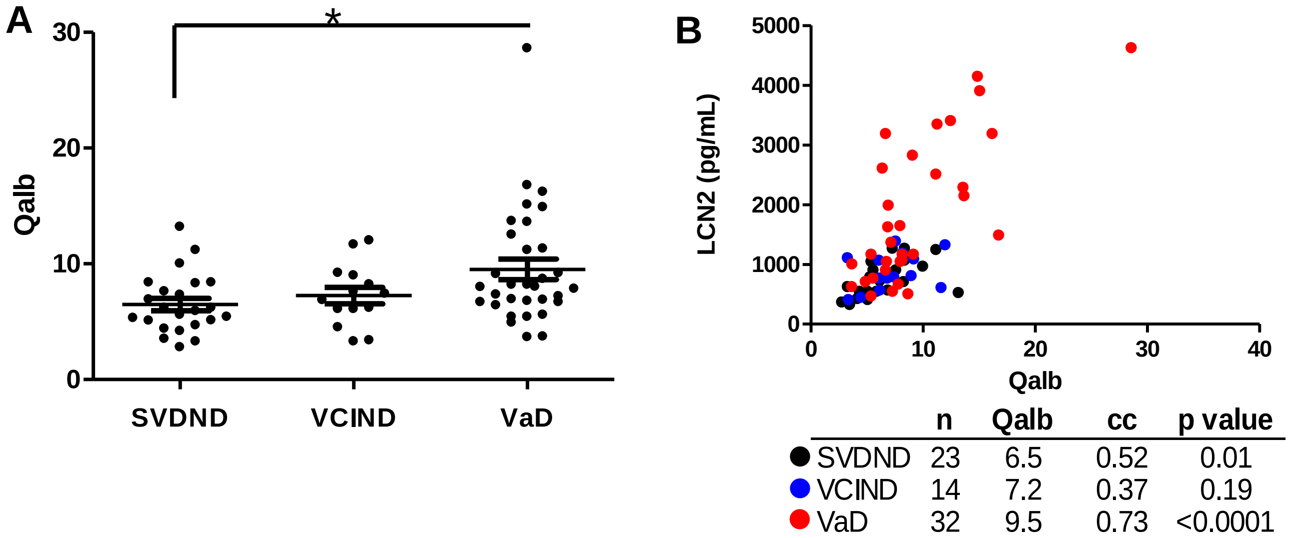

Association of CSF LCN2 with Qalb.

(A) CSF/Serum Albumin ratio (Qalb) in cerebral small vessel disease no dementia (SVDND), vascular cognitive impairment no dementia (VCIND), and vascular dementia (VaD) (cohort 1 and 4). Differences between groups, linear regression models controlled for age and sex. One way ANOVA with Bonferroni's post hoc, *p<0.05.

(B) Correlation analysis of Qalb and LCN2 in SVDND, VCIND, and VaD (cohort 1 and 4). Spearman correlation test, correlation coefficients (cc), and associated two-tailed p values.

Supplementary Figure 3.

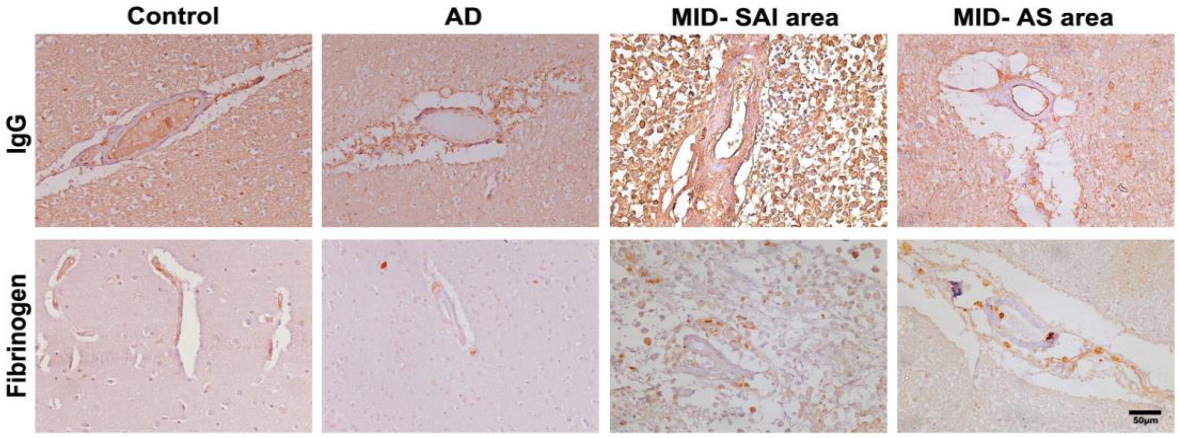

Blood vessel damage markers in the brain of AD and MID.

Blood vessel damage markers in cortex of Controls, Alzheimer's disease (AD) and Multi-Infarct Dementia (MID) in subacute infarct (SAI) and astrocytic scar (AS) areas. Immunohistochemistry with IgG (upper panels) and Fibrinogen (lower panels) are observed inside blood vessels in controls, while extravascular IgG and Fibrinogen deposits are observed in AD and MID cases. Paraffin sections counterstained with hematoxylin. Bar: 50µm.

Supplementary Table 1.

Comparisons of the AUCs between different cohorts

AUC cohort comparison	VaD vs. ND	VaD vs. AD
Cohort 1 vs. Cohort 2	0.405	0.669
Cohort 1 vs. Cohort 3	0.253	0.285
Cohort 2 vs. Cohort 3	0.799	0.627

Group discrimination between VaD and ND and discrimination between VaD and AD were considered, p-values of Z-test for Ho: AUC = 0.5 are shown.

Supplementary Table 2.

Differences in LCN2 concetrations between AD and VaD groups.

Cohort	p-value of the LCN2 difference between groups	p-value associated to MMSE as covariate
Cohort 1	0.0023	0.8682
Cohort 2	0.0059	0.9308
Cohort 3	0.0005	0.3492
Cohort 4	0.0131	0.3752

Significance in a statistical model controlling for the effect of age, sex and dementia. Tukey contrast p-value of the LCN2 difference and p-value associated to MMSE for Ho: $\beta_{MMSE} = 0$ score as covariate.

Supplementary Table 3.

Differences in LCN2 concetrations between AD and VaD groups.

Cohort	Pearson r	p-value
Cohort 1	0.12	0.04
Cohort 2	0.18	0.21
Cohort 3	0.21	0.16
Cohort 4	0.23	0.12

Pearson (LCN2 vs. age) correlation was applied to all cases. Correlation coefficients (r) and associated p-values are shown.
